# The relationship between high-sensitivity C-reactive protein and gallstones: a cross-sectional analysis

**DOI:** 10.3389/fmed.2024.1453129

**Published:** 2024-11-12

**Authors:** Zhimeng Jiang, Huixin Jiang, Xingyu Zhu, Donglin Zhao, Feifei Su

**Affiliations:** ^1^Graduate School of Hebei North University, Zhangjiakou, China; ^2^Department of Gastroenterology, Air Force Medical Center, Chinese People's Liberation Army, Beijing, China; ^3^School of Clinical Medicine, Haiyuan College of Kunming Medical University, Kunming, China; ^4^Department of Cardiovascular Medicine, Air Force Medical Center, Chinese People's Liberation Army, Beijing, China

**Keywords:** gallstones, cross-sectional study, high-sensitivity C-reactive protein, inflammatory, National Health and Nutrition Examination Survey

## Abstract

**Background and objective:**

High-sensitivity C-reactive protein (hs-CRP), a classical indicator of inflammation, holds significant clinical value in various diseases. The relationship between hs-CRP and gallstones, however, remains poorly studied at present. The relationship between hs-CRP and gallstones will be investigated in this study.

**Methods:**

Data from the 2017–2020 National Health and Nutrition Examination Survey (NHANES) were analyzed, focusing on participants aged 20 years and older who provided complete hs-CRP and gallstone information. Due to the skewed distribution of hs-CRP, the data were log-transformed [Log (hs-CRP)] to achieve normalization. Logistic regression analysis, subgroup analysis, and smoothed fitted curves were applied to determine the relationship between Log (hs-CRP) and the presence of gallstones.

**Results:**

The study included 4,484 participants with an average Log (hs-CRP) of 1.18 ± 0.74. The prevalence of gallstones was 11.15%, increasing with higher Log (hs-CRP) levels (quartile 1: 8.31%; quartile 2: 8.76%; quartile 3: 11.98%; quartile 4: 16.36%; *p* < 0.0001). Adjusting for all covariates in Model 3, each 10-fold increase in hs-CRP [corresponding to a one-unit increase in log10 (hs-CRP)] corresponded to a 29% increased odds of gallstones prevalence [1.29 (1.12–1.49)]. The smoothed fitted curve showed a positive linear relationship between Log (hs-CRP) and gallstones prevalence. The results of subgroup analyses exhibited a more pronounced positive correlation in the 20–40 age group [1.70 (1.33, 2.16)], compared to those aged 40–60 years [1.22 (1.01, 1.48)], and 60–80 years [1.14 (0.98, 1.34)].

**Conclusion:**

Higher Log (hs-CRP) levels are linked to a greater prevalence of gallstones. We still need to carry out further large prospective research to explore the causal relationship of this association.

## 1 Introduction

Gallstones refer to the presence of stones within the biliary system, including gallbladder stones and common bile duct stones, with cholesterol stones accounting for 80–90% of cholecystectomy cases (1). Epidemiological studies have shown a prevalence of 20–30% in European countries, 14–17% in the United States and 10–22% in India (2). Gallstones have become a significant health issue in developed countries, with their incidence rising annually, imposing a substantial burden on public health. The high costs of surgical treatment also place significant pressure on healthcare systems (3, 4). In the Third National Health and Nutrition Examination Survey (NHANES), over 14,000 representative samples from the United States underwent gallbladder ultrasonography, revealing that 1,149 individuals had gallstones. The prevalence was 8.6% in females, higher than the 5.3% observed in males (5). Most patients with gallstones are asymptomatic, with only a minority presenting with symptoms such as upper abdominal bloating and discomfort in the shoulder and back areas (6, 7). However, if gallbladder stones are accompanied by common bile duct stones, they may lead to a series of severe complications including acute obstructive suppurative cholangitis, acute pancreatitis, and obstructive jaundice. Without timely intervention, the condition can rapidly worsen, threatening the patient’s life and potentially resulting in liver failure and cholestatic cirrhosis (7–9).

C-reactive protein (CRP), an acute-phase protein secreted by the liver, is elicited in response to proinflammatory cytokines, often triggered by infection, inflammation, or tissue injury. Compared to the standard CRP assay, High-sensitivity C-Reactive Protein (hs-CRP) assay is more sensitive, capable of detecting lower levels of CRP, thereby providing a more precise assessment of a patient’s inflammatory status (10, 11). Metabolic disorders and inflammatory factors have been widely recognized as being associated with a wide range of chronic diseases, particularly in obesity and the metabolic syndrome, where inflammatory factors have a significant impact on disease formation (12, 13). Hs-CRP is associated not only with acute inflammation but also has been increasingly recognized as a highly sensitive biomarker for a range of conditions. Studies have demonstrated its significant clinical value in cardiovascular diseases, cancer prognosis, non-alcoholic fatty liver disease, obesity, and depression (14–19).

The formation of gallstones involves multiple risk factors such as female gender, obesity, metabolic syndrome, high cholesterol diet, estrogen levels, and genetic predisposition (20–22). Firstly, the development of oxidative stress within the gallbladder mucosa can result in the deterioration and damage of the gallbladder epithelial cells, leading to a reduction in gallbladder function and an increased propensity for the formation of cholesterol crystals (23). Secondly, inflammatory factors have the potential to impact the water and electrolyte balance in bile by influencing the absorptive and secretory functions of gallbladder epithelial cells, which may result in altered bile concentration and an increased risk of cholesterol deposits (24). Moreover, in animal studies, mice with concomitant cholesterol stones exhibited a markedly thickened mucus layer in the gallbladder. This thickened mucus layer impeded gallbladder emptying, thereby fostering an environment conducive to stone formation (25). Similarly, markedly elevated levels of circulating inflammatory proteins were observed in patients with gallstones (26), providing further evidence that inflammation plays a pivotal role in stone formation. Research indicates that elevated inflammatory markers may be risk factors for gallstone disease (27, 28). Several inflammatory indicators may be associated with gallstones in established NHANES studies. Despite hs-CRP being a classical biomarker for systemic inflammation, its relationship with gallstones has been minimally explored.

Consequently, we conducted an in-depth analysis utilizing data from the most recent NHANES (2017–2020) cycle to explore the relationship between hs-CRP and the gallstones in American adults. This study may provide a novel insight into the pathogenesis of gallstones for future research.

## 2 Methods

### 2.1 Study population

**Figure 1:**
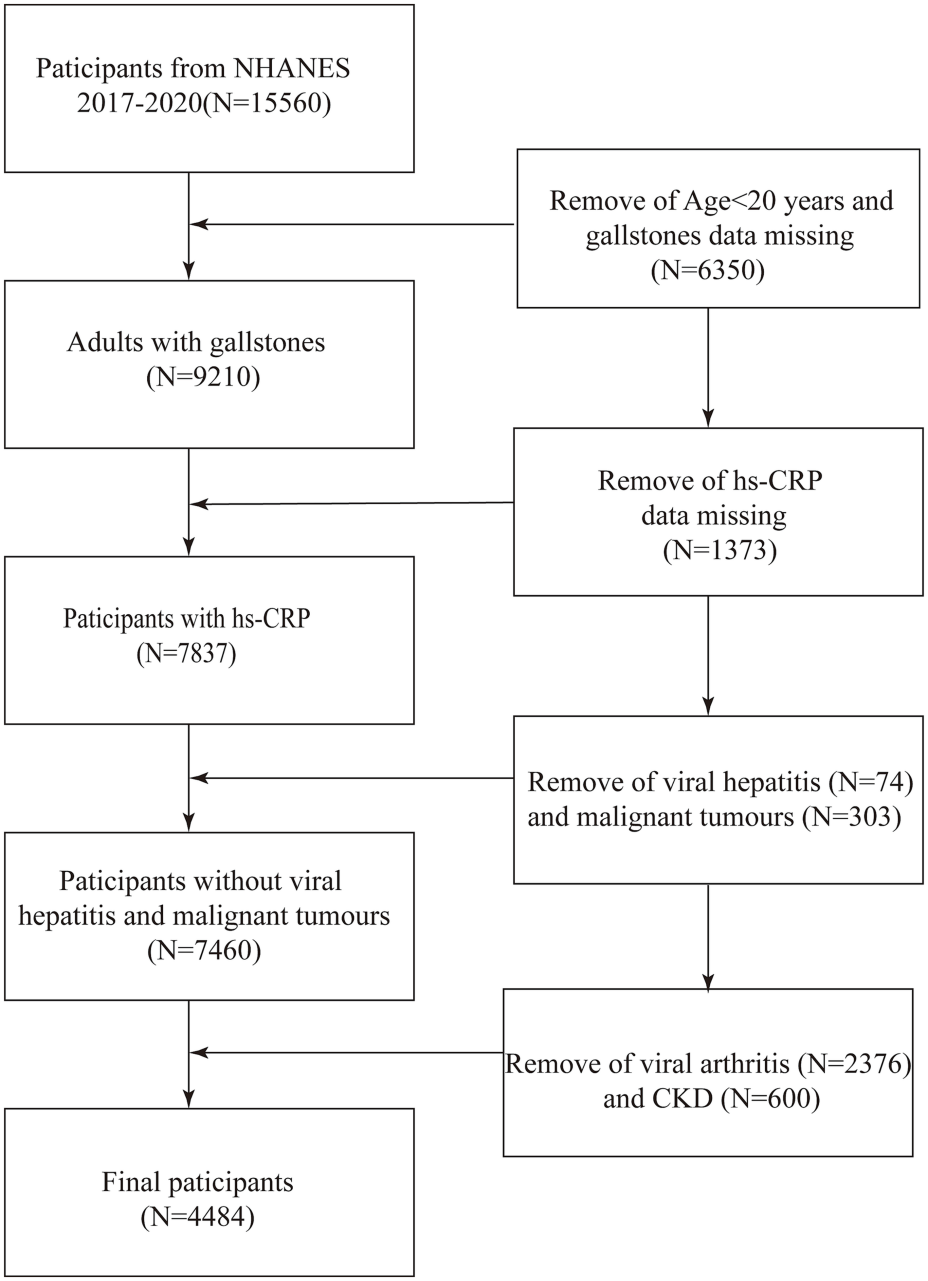
Flow diagram indicating study population. NHANES, National Health and Nutrition Examination Survey; Log (hs-CRP), Log-transformed High-sensitivity C-Reactive Protein; CKD, chronic kidney disease.

This study is a cross-sectional analysis designed to investigate the association between hs-CRP levels and the prevalence of gallstones. The present study utilized data from the NHANES, a continuous program initiated in 1999, currently provides the most up-to-date and accessible data up to 2020, designed to assess the health and nutritional status of adults and children in the United States. The study employed data from the NHANES for the years 2017–2020. As the data pertaining to gallstones is only available for participants aged 20 years and older, the study was focused on U.S. adults aged 20 years and older. Of the 15,560 individuals deemed eligible to participate, 6,350 were excluded due to their age being below 20 years and the absence of gallstone data. Additionally, 1,373 participants were excluded due to the unavailability of hs-CRP data. Furthermore, 74 subjects with a history of viral hepatitis, 303 subjects with a diagnosis of malignancy, 2,376 subjects with arthritis, and 600 subjects with chronic kidney disease, who may have had elevated hs-CRP levels, were also excluded. Ultimately, 4,484 individuals were enrolled in the study (Figure 1). The study protocol was approved by the Research Ethics Review Board at the National Center for Health Statistics. Prior to their involvement in the study, all participants were required to provide informed written consent.

### 2.2 Exposure variables and outcome definition

Hs-CRP levels were measured using a two-reagent, immunoturbidimetric system combined with a particle-enhanced immunoassay, with results reported in mg/L. The procedure involved initially incubating the sample with Tris buffer, followed by the addition of latex particles coated with mouse anti-human CRP antibodies. In the presence of circulating CRP, these particles aggregated, forming immune complexes that increased light scattering, directly proportional to the CRP concentration. The light absorbance generated was compared against a stored CRP standard curve to determine the concentration of CRP. Turbidity was measured at a primary wavelength of 546 nm, with a secondary wavelength of 800 nm. Serum specimens were processed, stored, and shipped to the Advanced Research and Diagnostic Laboratory at the University of Minnesota in Minneapolis for analysis. The lower limit of detection for hs-CRP was 0.15 mg/L. Preliminary analysis indicated a skewed distribution of hs-CRP data; thus, a log transformation [Log (hs-CRP)] was performed using base 10 to reduce variability and approximate a normal distribution. The outcome variable was gallstone incidence, assessed via self-reported survey responses to “Have you ever been told by a doctor that you have gallstones?” In the event that the participant indicated a positive response, the presence of gallstones was confirmed. The diagnosis of gallstones relied on self-reported data, which may introduce recall bias and affect the accuracy of the diagnosis.

### 2.3 Covariates

In the multivariable adjustment model, various potential confounding covariates were included: gender, age, race, poverty-income ratio (PIR), education level, marital status, drinking status, smoking status, moderate work activity, diabetes, high blood pressure (29, 30), total cholesterol, aspartate aminotransferase, alanine aminotransferase, high-density lipoprotein cholesterol, low-density lipoprotein cholesterol, white blood cell count, triglycerides, body mass index (BMI), and total calcium.

### 2.4 Statistical methods

NHANES employs a sophisticated multi-stage probability sampling design that incorporates stratification, whole cluster sampling, and weighting. All statistical analyses were performed in adherence to Centers for Disease Control and Prevention (CDC) guidelines, with the application of appropriate NHANES sampling weights to guarantee national representativeness of the resulting data. For continuous variables, variance-weighted analysis was applied, while weighted chi-square tests were utilized to evaluate group differences according to quartiles of Log (hs-CRP). Three models were constructed: Model 1 included no adjustments for covariates; Model 2 was adjusted to account for gender, age and race; and Model 3 accounted for all the study covariates. Sensitivity analyses and smoothed fitting curves were used to validate findings. Subgroup analyses were carried out according to gender, age, race, high blood pressure, diabetes, and BMI. *p*-values < 0.05 were considered significant. Sensitivity analyses and smoothed fitting curves were used to validate findings. Data were analyzed using Empower software^1^ and R version 4.1.3.

## 3 Results

### 3.1 Baseline characteristics

Table 1 presents a weighted distribution of clinical characteristics among participants stratified by quartiles of Log (hs-CRP) levels. The study included 4,484 adults, with an average age of 48.50 ± 17.33 years. Among the participants, 48.05% were male and 51.95% were female. Log (hs-CRP) quartile ranges were 0.10–0.60, 0.60–1.08, 1.08–1.69, and 1.69–4.41, respectively. Gallstones prevalence was 10.91%, with quartile-specific prevalence of 8.31, 8.76, 11.98, and 16.36%.

**Table 1:** Baseline characteristics of the study population.

Quartiles of Log (hs-CRP)					
Characteristic	Q1 (0.10–0.60)	Q2 (0.60–1.08)	Q3 (1.08–1.69)	Q4 (1.69–4.41)	*p-*value
Age (years)	46.20 ± 17.40	49.78 ± 17.42	49.99 ± 17.25	48.07 ± 16.92	<0.0001
Ratio of family income to poverty	3.34 ± 1.57	3.19 ± 1.55	2.99 ± 1.52	2.88 ± 1.57	<0.0001
White blood cell count (10^3^cells/μL)	6.47 ± 2.78	7.07 ± 1.96	7.41 ± 2.05	8.48 ± 8.60	<0.0001
Alanine aminotransferase (U/L)	20.08 ± 13.61	22.68 ± 16.14	23.94 ± 17.50	23.73 ± 19.12	<0.0001
Aspartate aminotransferase (U/L)	21.18 ± 10.31	21.58 ± 11.31	22.32 ± 13.40	22.13 ± 14.88	0.0477
Total bilirubin (mg/dL)	0.53 ± 0.31	0.49 ± 0.34	0.45 ± 0.28	0.40 ± 0.21	<0.0001
Triglycerides (mg/dL)	110.66 ± 73.72	140.12 ± 101.47	153.49 ± 105.28	158.26 ± 125.76	<0.0001
Cholesterol (mg/dL)	181.90 ± 39.97	187.58 ± 40.65	193.76 ± 42.51	187.75 ± 41.29	<0.0001
HDL-cholesterol (mg/dL)	59.44 ± 16.57	53.64 ± 16.33	51.77 ± 14.54	49.40 ± 14.08	<0.0001
LDL-cholesterol (mg/dL)	103.20 ± 34.76	108.96 ± 35.51	114.49 ± 37.72	109.99 ± 37.41	<0.0001
Total calcium (mg/dL)	9.31 ± 0.33	9.29 ± 0.35	9.32 ± 0.38	9.21 ± 0.37	<0.0001
Body mass index (kg/m^2^)	26.66 ± 7.24	26.30 ± 7.12	26.58 ± 7.55	26.82 ± 7.67	0.2747
Gender (%)					<0.0001
Male	52.05	54.60	47.01	36.91	
Female	47.95	45.40	52.99	63.09	
Race (%)					<0.0001
Mexican American	6.71	8.77	9.83	9.56	
Other Hispanic	6.22	6.76	8.13	9.28	
Non-Hispanic White	65.37	64.73	63.98	58.40	
Non-Hispanic Black	9.43	9.89	9.88	14.38	
Other race	12.27	9.85	8.18	8.38	
Education level (%)					0.0004
Less than high school	9.31	10.69	10.83	12.81	
High school	24.81	26.18	28.95	29.16	
More than high school	65.89	63.13	60.23	58.02	
Marriage (%)					0.8020
Yes	61.06	62.53	61.12	61.97	
No	38.94	37.47	38.88	38.03	
Smoking (%)					<0.0001
Yes	38.29	43.46	43.00	47.12	
No	61.71	56.54	57.00	52.88	
Drink (%)					0.0062
Yes	19.65	23.14	22.77	24.90	
No	80.35	76.86	77.23	75.10	
Moderate work activity (%)					0.6947
Yes	48.19	49.88	49.78	50.14	
No	51.81	50.12	50.22	49.86	
High blood pressure (%)					<0.0001
Yes	24.44	31.68	36.16	38.43	
No	75.56	68.32	63.84	61.57	
Diabetes (%)					<0.0001
Yes	6.73	8.77	11.76	18.14	
No	93.27	91.23	88.24	81.86	
Gallstones (%)					<0.0001
Yes	8.31	8.76	11.98	16.36	
No	91.69	91.24	88.02	83.64	

The Mean ± SD for continuous variables were calculated using a weighted linear regression model. For categorical variables, the probability value was calculated using a weighted chi-square test. Log (hs-CRP), log-transformed high-sensitivity C-reactive protein.

### 3.2 Higher levels of log (hs-CRP) are linked to a higher odds of gallstones prevalence

**Figure 2:**
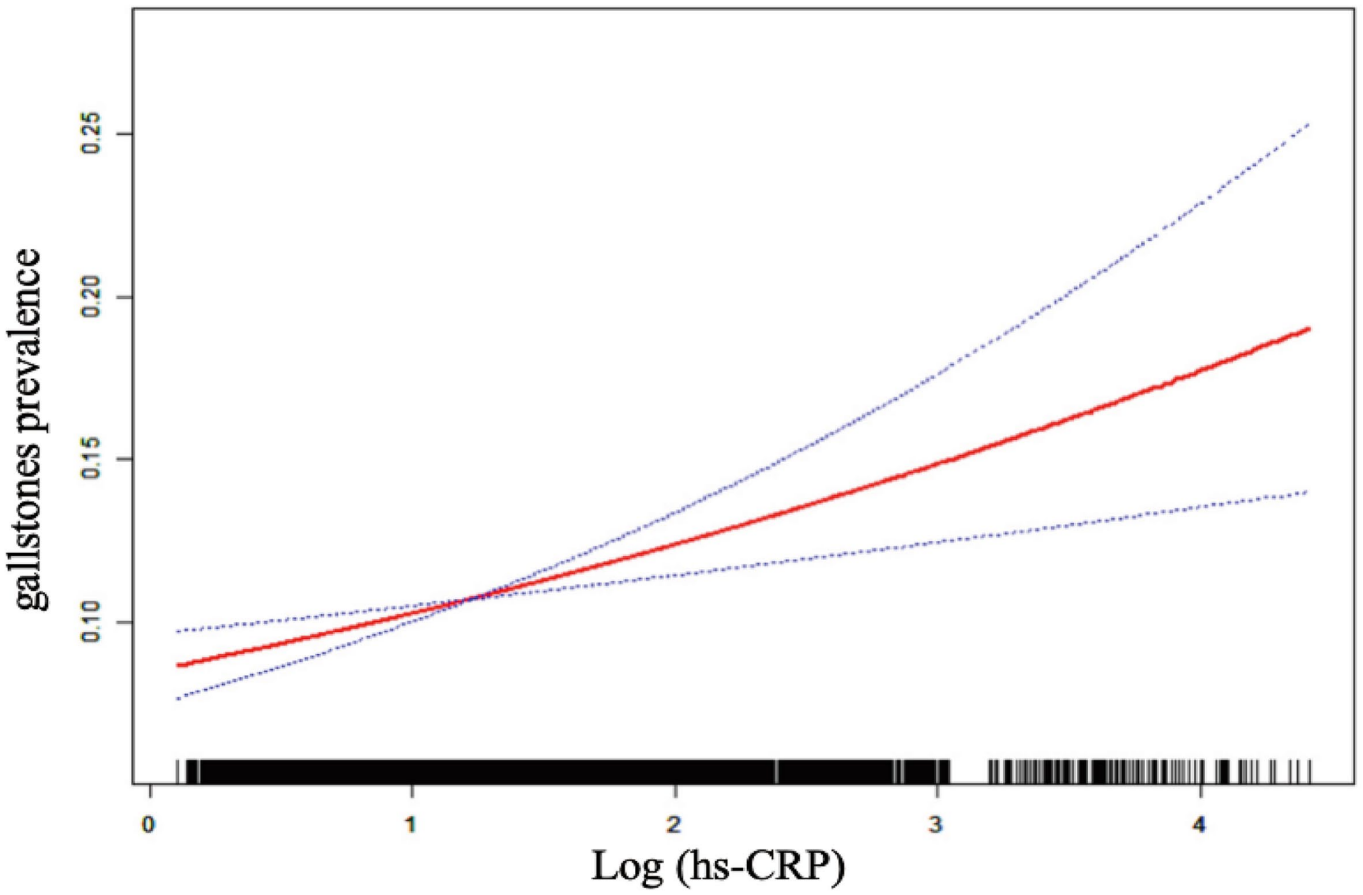
The linear associations between Log (hs-CRP) and gallstones. The solid red line illustrates the smooth curve fit between the variables. The blue bands represent the 95% confidence interval derived from the fit.

Table 2 demonstrates a positive correlation between Log (hs-CRP) and gallstones across all three models, with all the observed relationships being statistically significant. The effect measure used in this analysis is the odds ratio, which quantifies the odds of gallstones prevalence associated with hs-CRP. In Model 1, the positive correlation was found to be [1.44 (1.30, 1.59)], while in Model 2, it was found to be [1.38 (1.25, 1.54)]. After adjusting for all covariates, the positive correlation remained at in Model 3 [1.24 (1.11, 1.39)]. To further validate the stability of the correlation between Log (hs-CRP) and gallstones, we performed sensitivity analyses of Log (hs-CRP) from by interquartile level. The results demonstrated that this positive association remained consistent and statistically significant. Compared to the lowest quartile of Log (hs-CRP), the odds of gallstones prevalence in the highest quartile increased by 66% [1.66 (1.26, 2.20)]. The smoothed fitting curve results demonstrated a linear and positive relationship between Log (hs-CRP) and the prevalence of gallstones (Figure 2).

**Table 2:** Relationships between Log (hs-CRP) and gallstones.

Characteristic	Model 1 OR (95%CI)	Model 2 OR (95%CI)	Model 3 OR (95%CI)
Log (hs-CRP)	1.44 (1.30, 1.59)	1.38 (1.25, 1.54)	1.24 (1.11, 1.39)
Categories			
Q1	1.0	1.0	1.0
Q2	1.55 (1.19, 2.02)	1.36 (1.04, 1.78)	1.30 (0.99, 1.70) 0.0638
Q3	1.76 (1.36, 2.28)	1.52 (1.17, 1.98)	1.35 (1.03, 1.78) 0.0326
Q4	2.35 (1.84, 3.01)	2.07 (1.60, 2.68)	1.66 (1.26, 2.20) 0.0003
Log (hs-CRP) group trend	1.54 (1.36, 1.75) <0.0001	1.47 (1.29, 1.67) <0.0001	1.29 (1.12, 1.49) 0.0005

Model 1: no covariates were adjusted. Model 2: age, gender, and race were adjusted. Model 3: gender, age, race, PIR, educational level, marital status, drink status, smoking status, moderate work activity level, diabetes, high blood pressure, total cholesterol, aspartate aminotransferase, alanine aminotransferase, high-density lipoprotein cholesterol, low-density lipoprotein cholesterol, white blood cell count, triglycerides, BMI, and total calcium were adjusted. Abbreviation: Log (hs-CRP), log-transformed high-sensitivity C-reactive protein.

### 3.3 Subgroup analysis

Subgroup analyses were performed to explore the relationship between gallstone prevalence and the stability of Log (hs-CRP) across different population groups. Table 3 demonstrated a statistically significant positive correlation between Log (hs-CRP) and gallstone prevalence across nearly all subgroups. A comparison of age subgroup analysis revealed higher odds gallstones of prevalence among Americans aged 20–40 years [1.70 (1.33, 2.16)] compared to those aged 40–60 years [1.22 (1.01, 1.48)] and 60–80 years [1.14 (0.98, 1.34)], this association was found to be statistically significant, with an interaction *p*-value less than 0.05. The positive association between Log (hs-CRP) and gallstones remained consistent across gender, race, BMI, high blood pressure, and diabetes subgroups, with interaction *p*-values greater than 0.05.

**Table 3:** Subgroup analysis of the relationship between Log (hs-CRP) and gallstones.

Characteristic	Model OR (95%CI)	*p*-value	*P* for interaction
Stratified by age (years)			0.0226
20–40	1.70 (1.33, 2.16)	<0.0001	
40–60	1.22 (1.01, 1.48)	0.0366	
60–80	1.14 (0.98, 1.34)	0.0978	
Stratified by gender			0.1516
Male	1.12 (0.92, 1.37)	0.2532	
Female	1.33 (1.16, 1.52)	<0.0001	
Stratified by race			0.6067
Mexican American	1.37 (1.02, 1.84)	0.0363	
Other Hispanic	1.26 (0.88, 1.81)	0.2029	
Non-Hispanic White	1.27 (1.07, 1.52)	0.0063	
Non-Hispanic Black	1.33 (1.07, 1.65)	0.0105	
Other race	1.02 (0.75, 1.37)	0.9206	
Stratified by BMI			0.6113
Normal weight	1.32 (1.10, 1.59)	0.0032	
Overweight	1.18 (1.00, 1.40)	0.0498	
Obese	1.32 (1.06, 1.64)	0.0123	
Stratified by diabetes			0.0749
Yes	1.09 (0.92, 1.30)	0.3022	
No	1.31 (1.15, 1.50)	<0.0001	
Stratified by high blood pressure			0.5168
Yes	1.20 (1.03, 1.39)	0.0177	
No	1.28 (1.09, 1.51)	0.0028	

Model: gender, age, race, PIR, educational level, marital status, drink status, smoking status, moderate work activity level, diabetes, high blood pressure, total cholesterol, aspartate aminotransferase, alanine aminotransferase, high-density lipoprotein cholesterol, low-density lipoprotein cholesterol, white blood cell count, triglycerides, BMI, and total calcium were adjusted. BMI, body mass index (BMI), Log (hs-CRP), log-transformed high-sensitivity C-reactive protein.

## 4 Discussion

The results of the present study, a cross-sectional investigation involving 4,484 American adults, demonstrate a correlation between elevated Log (hs-CRP) values and an increased prevalence of gallstones. A linear positive relationship was observed between Log (hs-CRP) and gallstone prevalence, which remained stable even in the fully adjusted model. The results of our subgroup analysis revealed that the odds of gallstones prevalence associated with Log (hs-CRP) level were particularly elevated in the 20–40-year age group compared to other age groups.

To date, this is the inaugural cross-sectional study to look at the relationship between hs-CRP and gallstones in a US adult population. Previous research has indicated that gallstone prevalence is associated with inflammation. A cross-sectional study of 4,950 American adults revealed a positive correlation between the systemic immune-inflammation index and the increased odds of gallstones prevalence in individuals under the age of 50 (28). Another cross-sectional study examined the relationship between the dietary inflammation index and the composite dietary antioxidant index and the prevalence of gallstones, as well as the necessity for cholecystectomy. The results demonstrated a positive linear relationship between higher dietary inflammation index scores and gallstone prevalence, it also found that higher dietary inflammation index scores were associated with an earlier need for the first cholecystectomy (31). In comparison to the cohort study by Liu et al. (32), which explored the relationship between hs-CRP and gallstone risk in a Chinese population, our study extends these findings by examining a different population group—American adults. Our research adds to the evidence by demonstrating that this association is consistent across different ethnic groups. Moreover, while the Kailuan cohort study utilized a longitudinal design, which offers a higher level of evidence, our cross-sectional analysis complements this by providing a snapshot of this association in a diverse American population. In summary, our findings, when considered alongside Liu et al.’s results, suggest that this cross-population comparison highlights the potential universality of the relationship between inflammation and gallstone formation, thus strengthening the external validity of our findings.

Recent studies have shown that gallstone formation primarily involves bile acid metabolism, gallbladder function, and gut microbiota (22). The exact mechanism linking inflammation to the risk of gallstone disease remains unclear. However, the accumulating evidence indicates that inflammation is a pivotal factor in the formation of gallstones. The farnesoid X receptor (FXR) represents a class of nuclear receptors which exert a regulatory effect upon cholesterol and bile acid metabolism. Inflammatory responses may reduce the expression of nuclear receptors like FXR, affecting bile acid synthesis and secretion, thereby increasing cholesterol concentration in bile and promoting cholesterol crystal formation. In FXR knockout mice, the incidence of gallstones is significantly increased (33, 34). A study on the association between circulating inflammatory proteins and the risk of gallstones identified that higher levels of cytokines increased the risk of gallstones (27). Elevated levels of circulating inflammatory proteins and cytokines may induce inflammatory responses in gallbladder epithelial cells, leading to epithelial damage, fibrosis of the gallbladder wall, and reduced contractility of the gallbladder (24, 35, 36). Gallbladder motility disorders affect the contraction of the gallbladder and the expulsion of bile, creating favorable conditions for cholesterol nucleation and gallstone formation (37, 38). Another study found that DNA extruded from neutrophils and neutrophil extracellular traps act as adhesive agents binding calcium and cholesterol crystals, promoting the formation and development of gallstones (39).

In contrast with the conventional wisdom that age is a significant risk factor for gallstone formation (21, 22), our findings indicate that the prevalence of gallstones is markedly higher in younger individuals than in older age groups. This finding challenges the previous consensus that gallstone prevalence increases with age. The subgroup analysis demonstrates that there are higher 20–40 age group odds of gallstones prevalence compared to the 40–60 and 60–80 age groups, with a significant interaction between age and gallstone risk. For each 10-fold increase in hs-CRP [corresponding to a one-unit increase in log10 (hs-CRP)], the odds of gallstones prevalence increased by 70% in the 20–40 age group, 22% among those aged 40–60, and 14% in the 60–80 age group. It has been established by previous research that advanced age represents a significant risk factor for the development of gallstones. However, the results of our study do not align with this finding (40). We propose several possible explanations for this result: (1) Lifestyle factors: Young adults (20–40 years) may be more susceptible to engaging in unhealthy lifestyle habits, such as high-fat and high-sugar diets, which are known to increase the risk of gallstones (22). (2) Hormonal influences: This age group may also be influenced by changes in estrogen levels. Numerous studies have found that the incidence of gallstone formation is more prevalent in women (5, 41). One of the mechanisms through which estrogen may enhance the *in vivo* formation of cholesterol gallstones is by enhancing the efficacy of liver estrogen receptors (42, 43).

The results of our study indicate a markedly elevated risk of gallstone formation among individuals aged 20–40, particularly among those exhibiting elevated hs-CRP levels. This finding indicates that public health strategies should prioritize younger age groups, particularly those with unhealthy lifestyles or fluctuating hormone levels. The implementation of early screening and intervention strategies, such as dietary modifications and health education, may prove an effective means of preventing the formation of gallstones. Furthermore, hormone management for women should be a priority area of concern. Through early intervention, the incidence of gallstone-related complications can be reduced, as can the necessity for surgical treatment. This will result in a reduction in the economic burden on the healthcare system and yield long-term public health benefits.

Our study’s strengths include the use of NHANES data, managed by CDC, which ensures data rigor and authenticity through a nationally representative survey methodology. We also adjusted for numerous confounding variables, enhancing the validity and applicability of our findings. However, it is imperative to acknowledge the inherent limitations of the study. The diagnosis of gallstones relied on self-reported data from personal interviews, which introduces the potential for recall bias and inaccuracies in self-reporting. Additionally, the levels of hs-CRP, an inflammatory marker, may be influenced by a number of factors, including short-term infections, lifestyle changes, or other acute stressors. This can result in transient fluctuations, which may not accurately reflect the patient’s long-term or chronic inflammatory state, as a single measurement may not be sufficient to capture the full picture. Continuous measurements would provide a more robust assessment of the relationship between hs-CRP and gallstones. Furthermore, we did not account for the potential confounding effects of various medications that may influence hs-CRP levels or gallstone formation, such as statins or hormone replacement therapies. We also did not differentiate between types of gallstones (cholesterol vs. pigment stones), which could affect the relationship between inflammation and gallstone risk. Finally, despite the observation of associations, it was not possible to infer causality due to the cross-sectional study design employed. It is recommended that future studies adopt a more systematic approach in order to address the limitations of existing studies. Firstly, a comprehensive history of drug use should be obtained, encompassing all pertinent medications that may influence hs-CRP levels and gallstone formation, in order to account for potential confounding variables. Secondly, studies should differentiate between the various types of gallstones (e.g., cholesterol stones versus pigment stones), which would facilitate a more detailed exploration of the relationship between inflammatory markers and gallstone risk. Furthermore, the utilization of a longitudinal study design in conjunction with multiple hs-CRP measurements will enable investigators to more accurately capture changes in inflammatory status, thereby facilitating a more nuanced understanding of the causal relationship with gallstones. Such a study design will facilitate the provision of more practical guidance for clinical practice.

## 5 Conclusion

It has been demonstrated that higher Log (hs-CRP) levels are associated with greater odds of gallstone prevalence, particularly in younger adults. This observation highlights the need for future research to focus on the investigation of gallstone risk in younger populations. Our findings suggest that for individuals with elevated hs-CRP without a known cause, abdominal ultrasound screenings may help identify gallstones as a potential contributor. Early detection and intervention could reduce severe complications. hs-CRP may become an important reference index in future clinical practice for screening gallstones. However, further large prospective research is required to establish the causal relationship of this association.

## Data Availability

The datasets presented in this study can be found in online repositories. The names of the repository/repositories and accession number(s) can be found below: The survey data are publicly available on the internet for data users and researchers throughout the world (http://www.cdc.gov/nchs/nhanes/).

## Footnotes

^1^ http://www.empowerstats.com
