# Supplementary material for: The relationship between high-sensitivity C-reactive protein and gallstones: a cross-sectional analysis
Source: Front Med (Lausanne). 2024 Nov 12;11:1453129. doi: 10.3389/fmed.2024.1453129 (PMC11588438; doi:10.3389/fmed.2024.1453129)
Supplement: Supplementary file 1 [file Table_1.DOCX]

**Supplementary Table 1: Definition of covariables**

| **covariables** | **definitions of covariables** |
| --- | --- |
| Age | Range to 0-80, Age in years of the participant at the time of screening. Individuals 80 and over are topcoded at 80 years of age |
| Ratio of family income to poverty(PIR) | PIR was calculated by dividing total annual family (or individual) income by the poverty guidelines specific to the survey year |
| White blood cell count | White blood cell count were measured using a Beckman Coulter DxH 800 instrument |
| Alanine Aminotransferase | Alanine aminotransferase(ALT) is detected by catalysing the reaction of α-ketoglutarate with L-alanine to form L-glutamic acid and pyruvate, which is converted to lactate and NADH to NAD, with the decrease in NADH absorbance at 340 nm being directly proportional to the ALT activity |
| Aspartate Aminotransferase | Aspartate aminotransferase(AST) is measured by catalyzing the reaction of alpha-ketoglutarate with L-aspartate to form L-glutamate and oxaloacetate, where oxaloacetate is converted to malate and NADH to NAD, with the decrease in NADH absorbance at 340 nm being directly proportional to AST activity |
| Total Bilirubin | The method to measure total bilirubin is coupled with 3,5-dichlorophenyl diazonium in the presence of a solubilizing agent in a strongly acidic medium. The intensity of the red azo dye formed is directly proportional to the total bilirubin and can be determined photometrically (546 nm) |
| Triglycerides | Triglycerides are hydrolysed to glycerol by microbial lipoprotein lipase and then oxidised to produce hydrogen peroxide which forms a red dye under the action of peroxidase, the intensity of the colour is proportional to the concentration of triglycerides and can be measured photometrically |
| Cholesterol | Cholesterol is measured enzymatically by converting esterified cholesterol to free cholesterol via cholesterol esterase, followed by oxidation with cholesterol oxidase to produce cholest-4-en-3-one and hydrogen peroxide, which then reacts with 4-aminophenazone and peroxidase to form a coloured product that can be measured at a wavelength of 505 nm |
| HDL-Cholesterol | Specific measurement of HDL-Cholesterol is achieved by PEG-modified cholesterol enzymes reacting preferentially with HDL-Cholesterol under specific conditions to produce a pigment detectable at 600 nm |
| LDL-Cholesterol | The Roche method of LDH measurement is derived from the formulation recommended by the International Federation of Clinical Chemistry |
| Total Calcium | Total calcium was measured by reacting total calcium with 5-nitro-5'-methyl-BAPTA under alkaline conditions to form a complex, which then reacted with Ethylenediaminetetraacetic acid (EDTA) to form a coloured product, which was measured photometrically at 340 nm |
| Body Mass Index | Body Mass Index was calculated as weight in kilograms divided by height in meters squared, and then rounded to one decimal place |
| Gender | Both males or females |
| Race | The race variable comes from responses to survey questions about race and Hispanic origin, including mexican american, other hispanic, non-hispanic white, non-hispanic black, and other race |
| Education level | This variable is the highest grade or level of education completed by adults 20 years and older, inlcuding less than high school, high school, and more than high school |
| Marriage | Marriage is defined as married or unmarried |
| Smoking | Smoking is defined as at least 100 cigarettes in a lifetime |
| Drink | Drink is defined as 4/5 cups or more of alcohol per day |
| Moderate work activity | Moderate work activity was defined as whether the subject had an activity that would result in a small increase in respiration or heart rate, such as brisk walking for at least 10 consecutive minutes or carrying light objects? YES or No |
| High blood pressure | Ever told you had high blood pressure? Yes or No |
| Diabetes | Did your doctor ever tell you you had diabetes? Yes or No |

Abbreviation: NADH, Nicotinamide adenine dinucleotide + hydrogen. NAD, Nicotinamide adenine dinucleotide
